# Users' Acceptability and Perceived Efficacy of mHealth for Opioid Use Disorder: Scoping Review

**DOI:** 10.2196/49751

**Published:** 2024-04-11

**Authors:** Lynnette Nathalie Lyzwinski, Mohamed Elgendi, Carlo Menon

**Affiliations:** 1 Menrva Research Group, School of Mechatronics Systems Engineering and Engineering Science Simon Fraser University Vancouver, BC Canada; 2 ETH Biomedical and Mobile Health Technology Lab Zurich Switzerland

**Keywords:** acceptability, addict, addiction, addictions, app, app-based, application, applications, apps, barrier, barriers, challenge, challenges, messaging, mHealth, mobile health, monitoring, opioid, opioids, overdose, overdosing, pharmacology, review methodology, review methods, scoping, sensor, sensors, SMS, substance abuse, substance use, text message, wearable technology, wearable, wearables

## Abstract

**Background:**

The opioid crisis continues to pose significant challenges to global public health, necessitating the development of novel interventions to support individuals in managing their substance use and preventing overdose-related deaths. Mobile health (mHealth), as a promising platform for addressing opioid use disorder, requires a comprehensive understanding of user perspectives to minimize barriers to care and optimize the benefits of mHealth interventions.

**Objective:**

This study aims to synthesize qualitative insights into opioid users’ acceptability and perceived efficacy of mHealth and wearable technologies for opioid use disorder.

**Methods:**

A scoping review of PubMed (MEDLINE) and Google Scholar databases was conducted to identify research on opioid user perspectives concerning mHealth-assisted interventions, including wearable sensors, SMS text messaging, and app-based technology.

**Results:**

Overall, users demonstrate a high willingness to engage with mHealth interventions to prevent overdose-related deaths and manage opioid use. Users perceive mHealth as an opportunity to access care and desire the involvement of trusted health care professionals in these technologies. User comfort with wearing opioid sensors emerged as a significant factor. Personally tailored content, social support, and encouragement are preferred by users. Privacy concerns and limited access to technology pose barriers to care.

**Conclusions:**

To maximize benefits and minimize risks for users, it is crucial to implement robust privacy measures, provide comprehensive user training, integrate behavior change techniques, offer professional and peer support, deliver tailored messages, incorporate behavior change theories, assess readiness for change, design stigma-reducing apps, use visual elements, and conduct user-focused research for effective opioid management in mHealth interventions. mHealth demonstrates considerable potential as a tool for addressing opioid use disorder and preventing overdose-related deaths, given the high acceptability and perceived benefits reported by users.

## Introduction

### Overview

Since the COVID-19 pandemic, the incidence of opioid overdose has increased significantly among young adults [1,2]. According to research conducted in Canada, there was a 135% increase in opioid overdose–related deaths per week in Ontario during COVID-19 in comparison to prepandemic times [2]. Excess levels of opioids may lead to respiratory depression and cardiopulmonary failure, resulting in loss of consciousness and death [3,4]. It is treated with naloxone, an antagonist that prevents opioid-related fatalities [5,6]. Thus, it is essential to find ways to manage opioid use disorder (OUD) and prevent overdose. A study reviewing smartphone apps that were commercially available until 2019 found that there was a lack of evidence on their ability to be useful for monitoring OUD [7]. The study’s authors also reviewed interventional studies involving mobile health (mHealth) for opioid use; most of the reviewed studies included smartphone apps or personal digital assistants, and only a minority of those devices had a wearable biosensor [7]. The authors concluded that there is a gap in the literature and that more studies are needed to address OUD [7].

Since that study was published, there have been technological developments in this area to meet the pressing need that arose during the COVID-19 pandemic. Specifically, new wearable sensors have been developed that monitor sweat, heart rate, and temperature and that predict overdose in patients [7-13]. Several studies have evaluated the application of wearable opioid sensors for detecting overdose by detecting changes in temperature, movement, respiratory rate, heart rate, and electrodermal activity [8,11-17]. Most of the studies involved a wrist-worn biosensor, usually the Empatica E4 biosensor and Q sensor, which integrate a machine learning algorithm. A few studies found that wearable technology can detect changes in skin temperature, notably a temperature rise during opioid intake and overdose [8,12-14,16,17]. Carreiro et al [14] found that the mean skin temperature increase after opioid intake was 2.62 °C and that differences in body temperature before and after intake were significant (*P*<.01). In addition to temperature changes, several studies found reduced movement or locomotion by evaluating triaxis acceleration data [8,11,13,14,16,17]. Notably, local extremities, such as the fingers, had reduced motion or there was less “fidgeting” after opioid intake. In 1 study, fidgeting was observed more in heavy opioid users than less heavy users [14]. Emerging research also suggests that these devices may be valid for detecting opioid intake and overdose [8,11,16]. For example, Mahmud et al [11] found that the Q Affectiva sensor had an accuracy of 99% for detecting opioid intake in users.

Although emerging technological research in the wearables and mHealth domain suggests that these devices may be promising for detecting opioid overdose and assisting with OUD, little is presently known about the acceptability of this form of monitoring from the patients’ perspectives. In other words, a review of qualitative studies on consumer perspectives has not been undertaken. While a device may work in theory, it may fail to be an effective intervention and be implemented within the community care setting if the technology is not acceptable for opioid users or if they do not adhere to the technological intervention. While the quantitative efficacy of these devices has been established in a past review [7], there is a need to understand how usable these devices are for patients. Often, many patients with substance use disorders are difficult to reach and treat due to marginalization or stigma [18-20], making them resistant to accessing medical help [21]. Thus, there is a compelling research interest to determine how patients and opioid users feel about the emerging technological developments that may assist them in managing their substance use and preventing overdose fatalities.

### Aims

This study aimed to undertake a review of opioid users’ perspectives on mHealth and wearable technology for OUD and overdose management, with a focus on the latest technology developed over the last 5 years. We aimed to better understand whether users found the technology acceptable to use and helpful. We also aimed to better understand the perceived barriers as well as the benefits of using wearables for OUD and opioid overdose management among opioid-using populations. This study focused on qualitative research in order to better understand consumer experiences with wearable technology for OUD, with the overarching goal of making recommendations for future technological development and intervention design.

## Methods

### Study Guidelines

A scoping review was performed to summarize the use of wearable devices in managing OUD to better understand the key benefits, preferences, and barriers of use. The guidelines of the PRISMA-ScR (Preferred Reporting Items for Systematic Reviews and Meta-Analyses extension for Scoping Reviews) [22] were followed.

### Search Strategy and Study Eligibility

The PubMed (MEDLINE) and Google Scholar databases were searched for all studies published until July 2022 using the search strategy in Textbox 1.

**The search strategy used to search for studies.**

“Wearable Electronic Devices”[Mesh] OR wearable*[tiab] OR “Smart Band*”[tiab] OR “Smart Watch*”[tiab] OR mHealth OR “mobile health” OR app OR application OR wristband OR ((sensor*[tiab] OR sensing[tiab] OR biosensor*[tiab]) AND (wear*[tiab] OR worn[tiab]))

((“Analgesics, Opioid”[Mesh] OR “Analgesics, Opioid” [Pharmacological Action] OR Opiate*[TIAB] OR Opioid*[TIAB] OR “fentanyl”[tiab] OR “hydromorphone”[tiab] OR “meperidine”[tiab] OR “morphine”[tiab] OR “oxycodone”[tiab] OR “pentazocine”[tiab] OR “sufentanil”[tiab] OR “tramadol”[tiab] OR “morphine”[MeSH Terms] OR “hydrocodone”[MeSH Terms] OR “hydrocodone”[tiab] OR “buprenorphine”[MeSH Terms] OR “buprenorphine”[tiab] OR “codeine “[MeSH Terms] OR “fentanyl”[MeSH Terms] OR “hydromorphone”[MeSH Terms] OR “meperidine”[MeSH Terms] OR “oxycodone”[MeSH Terms] OR “pentazocine”[MeSH Terms] OR “sufentanil”[MeSH Terms] OR “tramadol”[MeSH Terms] OR OxyContin[tiab] OR Vicodin[tiab] OR “Codeine”[Mesh] OR codeine[tiab] OR morphine[tiab]) AND (“substance use disorder”[tiab] OR “substance abuse”[tiab] OR disorder[tiab] OR abuse[tiab] OR use[tiab])) OR “Opioid-Related Disorders”[Mesh] OR “Opiate use disorder”[tiab] OR “opioid use disorder”[tiab] OR “opiate dependence”[tiab] OR “opioid dependence”[tiab] OR “opiate abuse”[tiab] OR “opioid abuse”[tiab]

### Inclusion and Exclusion Criteria

All qualitative studies that evaluated opioid users’ perspectives on the latest mHealth wearable devices or portable mHealth devices (including apps and SMS text messaging–based interventions) for monitoring opioid use were included in the review. Quantitative studies were only included if they had a qualitative component or if they had collected more detailed information on acceptability, benefits, and barriers associated with use. The technology must have been emerging and recently developed (within the past 5 years, with a focus on technology during the pandemic). Web-based interventions were excluded if they did not include a wearable or mHealth element.

### Data Screening and Extraction

Data were screened according to titles, and abstracts, and followed by full texts of articles meeting inclusion criteria. The 2 reviewers, LNL and ME, screened the articles for inclusion and met when there was disagreement. Data on user perspectives, including benefits, barriers, and preferences, were extracted and summarized in tabular format. General study characteristics, including location, methods, participant age, and gender, as well as the type of technology, were also extracted.

## Results

### Overview

**Figure 1:**
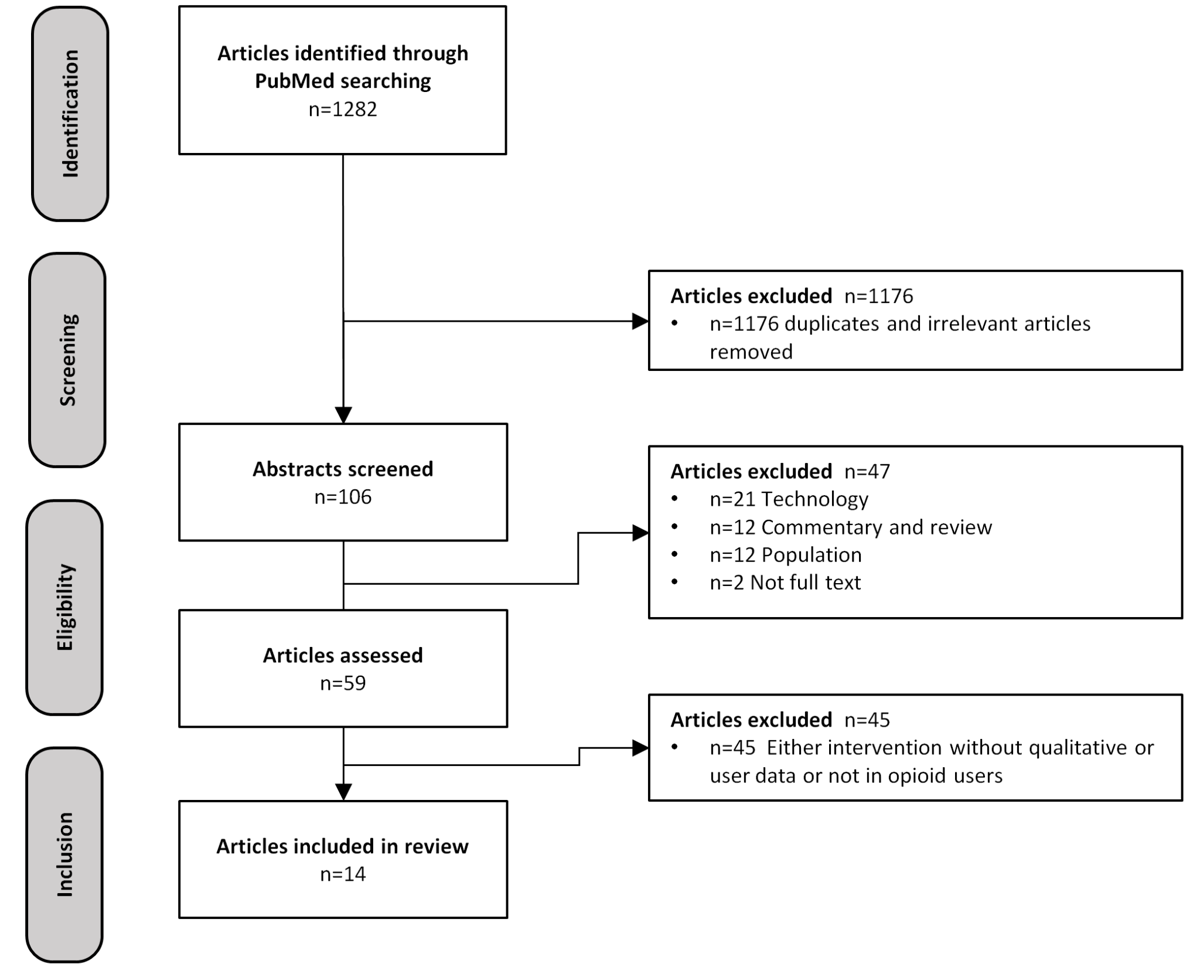
PRISMA (Preferred Reporting Items for Systematic Reviews and Meta-Analyses) flowchart of the study.

A total of 14 studies met the criteria and were included in the review [9,23-35]. Details of the search and stages of selection are outlined in Figure 1. Most of the studies were undertaken in the United States. One study was undertaken in Singapore and another in Canada. Most of the studies were relatively evenly distributed by sex, and most participants were middle-aged.

### Usability, Feasibility, and Acceptability

Overall, the studies that explored the usability and acceptability of mHealth technology for opioid monitoring found relatively high usability and acceptability among the participants. A summary of the key issues and perspectives raised by the participants with OUD is described in Table 1. In Waselewski et al’s [34] study, the mean useability score was 86.9 in the participants using opioids; in health care providers, the score was also high at 83.3. Another study found that 71% of the patients had a high level of satisfaction when using a mobile app with telemedicine built-in videos to monitor medication use [23]. In that study, the usability was also high; 93% of the patients did not report having had any technical issues, and 72% submitted their videos using the app [23]. Similarly, a wearable biosensor for overdose monitoring also had a high level of support from participants and a willingness to wear it [25]. Although the study by Hawk et al [24] had a high initial survey response rate (95%), which highlights interest among opioid users, completion of the surveys declined over the course of 1 month, from 97% at baseline to 42% at 4 weeks. Kanter et al [9] found that patients were willing to wear the device to monitor opioid overdose all the time (76%). Most patients were willing to share personal data on their opioid intake [24,31]. For example, in 1 study, 81% of the patients were willing to use the mHealth device to help them taper off opioids [31]. Comfort was also identified as an important factor in determining acceptability and wearability for participants in 2 studies [9,24].

**Table 1:** Study characteristics.

Study; country; type of study	Number of participants (M%^a^ and F%^b^); Age	Technology	Procedure and measures	Results
Waselewski et al (2021) [34]; United States; 6-month pilot with qualitative interview	25 (52% and 48%); mean 33.7 (SD 8.1) years	Hope app for opioid user disorder	Feasibility, usability, and acceptability	Patient Usability: mean score 86.9 (SD 10.2) Providers: mean 83.3 (SD 12.8) Patients liked self-monitoring, enhanced support, communication, and contact with providers
Godersky et al (2020) [23]; United States; N/A^c^	14 (86% and 14%); 18-65 years	mHealth^d^ app for opioid and telemedicine (observation of buprenorphine intake) use in the office	Feasibility and acceptability	93% had no problems with using the app to share videos Adherence= 72% High satisfaction= 71 % of users Pros: simplicity of use, structure, and personal accountability Cons: self-portrayal when recording
Hawk et al (2021) [24]; United States; pilot feasibility	101 (43.7% and 57.3%); mean 38.4 (SD 10.25) years	mHealth platform reporting patient outcomes	Feasibility and acceptability	Registration=95% Declining rate of completion of surveys over time: Day 1=97% Day 3= 49% Month 1=42% Willingness of patients to share data on medication, visits, substance use. Barriers: Wi-Fi connection and access to technology Email retention issues mHealth log-in information retainment patient factors: comfort Privacy is a concern in a minority
Kanter et al (2021) [9]; United States; cross-sectional study with semistructured interviews	97 (57% and 43%); mean 41 (range 37-49) years	Wearable device for opioid detection, overdose, and treatment	Acceptability or likability and willingness to use (usability)	Theme: privacy or discreetness is important Theme: comfort when wearing it Theme: a device that tracks and helps with overdose is needed Willingness to use an mHealth device for opioid overdose=76% Wear device continuously=75.5% Vital sign monitoring=77% Alerts others of overdose=63% Watch type of bracelet design preferred=77% Necklace=51% Advised giving this to the hardest to reach and treat (eg, opioid intolerant or homeless)
Magee et al (2021) [31]; Australia; N/A	21 (48% and 52%); N/A	mHealth SMS text message supportive intervention	Qualitative acceptability and willingness to use	Willingness of patients to engage with SMS text messaging to help curtail opioid use (from prescriptions)=81% App-based support=71% Desire for education and social support (socioeconomic support) Barriers: internet; access to technology; visual problems; phone signal strength; poor self-assurance for knowledge around technology such as apps Adherence could be improved through greater integrated medical or physician care, flexibility with dosing, and regular replies Pain management advice Need for support from a person as well Need for encouragement and motivation
Marcu et al (2020) [32]; United States; N/A	19 (53% and 47%); 18-36 years	Smartphone app for opioid overdose	Acceptability or likability and user perspective	Positive: mHealth can increase accessibility to care including medical supportive therapy for preventing overdose fatalities Negative: privacy concerns, geolocation tracking of apps, and theft, and private data in apps
Ahamad et al (2019) [25]; Canada; N/A	1061 (63.1% and 36.9%); median 44.2 years	Wearable opioid biosensor	Factors linked with willingness to wear the device	Barriers: homelessness High willingness to wear the opioid sensor Predictors of openness to try the sensor: Higher in women (aOR^e^=1.41; 95% CI 1.09-1.84) Those who were on methadone treatment regimes (aOR=1.86; 95% CI 1.45-2.40) and history of overdose (aOR 1.39; 95% CI 1.06-1.83)
Eaves et al (2022) [26]; United States; N/A	27 (0% and 100%); N/A	mHealth app for substance abuse (general opioid and polysubstance abuse, alcohol)	Qualitative 5 focus groups	Desire to access social services: housing and counseling parenting help Respectful safe environment Supportive tone in SMS text messages, connect with social support (peers) and health professionals Did not want the app to feel like a chore (eg, points earned)
Flickinger et al (2022) [27]; United States; N/A	28 (52% and 48%); mean 33.7 (SD 8.1) years	Hope app and community message board	User engagement and conversation in the app	Opioid users exchanged supportive messages with one another and physicians through the app They asked for medical advice=52% Social support subjects=8% App related interaction=45%
Glass et al (2021) [28]; United States; N/A	14 (71% and 29%); mean 40.1 (19-65) years	mHealth apps (opioid and cannabis users)	Semistructured interviews	Apps should be a part of the primary care encounter and communication Endorsement from medical professionals Support and interaction with physicians, instructing them SMS text messaging and professional health support through phone Convenience Minimize hassle Trust essential
Hodges et al (2022) [29]; United States; pilot study	25 (52% and 48%); mean 34 years	Hope app	Self-efficacy and engagement	Loss to follow-up a problem due to distance mHealth app usage continued Reported increases in self-efficacy to abstain
Tofighi et al (2022) [33]; United States; N/A	50 (48% and 52%); mean 44.1 (SD 11.8) years	SMS text message through texting software (ApToto)	Analysis of text messages	Desire for personally tailored messages Frequency according to risk profiles Professional health support Video-based content and advice 6% opted out of messages Reply to SMS text message over month=88% Buprenorphine advice=2% messages Cognitive behavioral therapy–based replies=13.8% Appointments=6.1%
Zhang et al (2019) [35]; Singapore; N/A	30 (66.7% and 33.3%); mean 47.9 (SD 11.8) years	mHealth smartphone intervention for polysubstance (opioid, cannabis)	Thematic analysis and surveys	App was liked and motivating=54% Self-efficacy or confidence=33% Interactive=77% Easy=100%
Langdon et al (2021) [30]; United States; N/A	24 (50% and 50%); mean 38.9 years	Digital health interventions, computer, and SMS text message–based opioid users	Semistructured interviews on preferences	Motivational element is essential Distress tolerance Assists with learning Reduces stigma or being judged Automated and tailored messages Frequency=2-3 messages per day Engaging use of media, videos (links), emojis, and GIFs

^a^M%: percentage of male individuals.

^b^F%: percentage of female individuals.

^c^N/A: not available.

^d^mHealth: mobile health.

^e^aOR: adjusted odds ratio.

### Perceived Benefits

The synthesis of qualitative research reveals shared advantages among participants regarding mHealth technologies for opioid management. Accessibility stands out as a key benefit, with an emphasis on the pivotal role of behavior change. For example, 1 theme was that the opioid mHealth wearable tracker could provide enhanced access to care, which was perceived to be needed [31,32,34]. This included greater contact with health care services and medical providers [32,34]. These trackers, particularly wearable forms such as bracelets, wristbands, and necklaces, were favored for their noninvasive nature, as mentioned in Kanter et al [9]. Participants in 2 studies recognized a clear need for an opioid monitoring device that would help prevent overdose and fatalities [9,32], echoing the sentiment for an effective opioid monitoring tool. For example, the study by Kanter et al [9] found that 77% of patients reported that wearables were beneficial for monitoring vital signs, while 63% saw their value for alerting bystanders of an overdose. Self-monitoring and accountability were brought up as benefits in 2 studies [23,34]. Convenience and ease of use were also identified as benefits of using wearables for opioid use management [23,28,35]. A total of 2 studies found that a mHealth intervention increased the self-efficacy of opioid users and polysubstance users to overcome substance abuse [29,35], and motivation was brought up as a benefit as well [30,35]. These findings collectively underscore the value of mHealth apps in supporting individuals with OUD through various practical and psychological avenues.

### Perceived Barriers

The research across various studies delineates several challenges in the adoption and continuous use of mHealth technologies for opioid management. Privacy stands out prominently as a concern. The apprehension over safeguarding personal health information was paramount, particularly with wearables that track opioid use. Protecting privacy and personal information was one of the main concerns participants had with tracking opioid use through wearable devices [9,32]. Privacy was mainly related to protecting personal and private health information. This included geolocation tracking as well as theft of the device if the patient was unconscious from an overdose [32] as well as concerns with the privacy of electronic health records and log-in information retention [24].

The visibility of the device and the need for it to be inconspicuous during use were significant factors in Kanter et al’s [9] study, while Godersky et al [23] identified discomfort with self-viewing during video recordings as a deterrent. These issues underscore the need for privacy in both data handling and physical device usage.

Technological barriers also emerged, with several studies pointing to issues such as unreliable phone reception, limited access to necessary hardware, and inconsistent Wi-Fi connections, as indicated by Hawk et al [24]. These issues are compounded by the digital literacy required to effectively use such apps, with some users lacking the confidence to navigate mHealth technology, as might be inferred from some studies [24,31].

Visual difficulties in interacting with devices were also noted as a hindrance in 1 study [31]. Ahamad et al [25] report that homelessness can be a substantial obstacle to the effective use of wearable biosensors for overdose monitoring. Moreover, Kanter et al [9] suggest that interventions should aim to reach the most vulnerable populations who stand to benefit significantly from mHealth solutions.

The importance of human support in conjunction with technological aids was also mentioned, indicating that personal interaction may bolster adherence to using mHealth apps, a sentiment that could be associated with the insights from Magee et al [31]. These findings highlight the multifaceted nature of the barriers faced and the need for a holistic approach to mHealth implementation for OUD management.

### Preferences

The research presents a range of preferences by participants when engaging with mHealth technologies for opioid management. Central to these preferences is the desire for behavior change facilitation through various techniques. The main behavior change techniques (BCTs) that were preferred by participants across the studies included encouragement or motivation [30,31], social and personal support [26,27,31,34], professional support [28,31], education [31,35], and personal tailoring [30,33]. Most notably, participants expressed a desire to have contact with a health care professional and to receive encouragement and educational, motivational, socioeconomic, and social support [26,31,33]. With regard to professional help, some participants wanted to have more medical professionals on board with the app rather than requiring users to navigate the app without professional support [26,28]. Integrated medical care from providers when using the wearables was preferred by participants in 5 studies [26,28,31-33]. For example, the study by Glass et al [28] found that participants preferred to have conversations with their medical providers about mHealth apps as part of their treatment plan. Another study found that 52% of users asked for medical advice from health practitioners while using the app [27].

In addition to this, peer social support was highlighted as being important in 2 studies [26,27], with app-based support being preferred by 71% of participants in another study [31]. Participants in 1 study wanted the mHealth app to integrate social assistance, housing services, counseling, and medical and peer support into the application and treatment regime [26].

Tone was also brought up as a theme in 1 study. Specifically, participants preferred to receive messages with a supportive and respectful tone; moreover, they wanted the technology to feel safe to use [26]. Integration of visual elements, including emojis, video links, and memes, was identified as being important in 1 SMS text messaging study [33]. Trust was an important factor in 2 studies [26,28]. Discreetness was essential, as was the need for mHealth to reduce the stigma associated with substance use [30,32]. In addition to automated messages, personally tailored messages were preferred by the participants in 2 studies [30,33]. The need for motivation and encouragement was brought up as an important theme in 2 studies [30,31].

Additionally, 2 studies found that participants desired the app to be hassle-free or to not feel like a tiresome exercise to complete [26,28]. The personalization of messages and ease of use are underscored, with participants favoring tailored communication as seen in studies [30,33], and a seamless, nonburdensome interaction with the technology as suggested in 2 studies [26,28]. These preferences highlight the need for mHealth interventions to be user-centered, providing not only the technology but also the necessary human and educational resources to support individuals with OUD.

## Discussion

### Overview

This study aimed to better understand the perspectives of consumers of wearable technology and mHealth apps for detecting opioid intake and overdose in patients with OUD. Overall, it appears that patients are open to using mHealth technology and wearable devices to help manage opioid-related substance use and detect overdose. The common themes around the ability of technology to save lives and increase access to health care were a benefit that was noted in a few studies. Comfortable wearable sensors also appeared to be important as was technology that protected the user’s privacy. Special attention must be paid to protecting the privacy of opioid users, as this was brought up as a concern and a potential disadvantage of the emerging technological interventions. Indeed, patient privacy and data protection are key hallmarks of the ethical principles surrounding modern health technology. Privacy must be protected to prevent unauthorized access to sensitive and personal information.

To manage the technological barriers associated with mHealth apps and wearable devices, users should be trained and reassured of their safety. This will increase their confidence in their ability to use this type of technology to manage their substance use condition. As users in 1 study wanted encouragement and motivation, these BCTs [36] should be incorporated in future interventions [31]. Other BCTs that may be considered include integrating social and professional health support and sending reminders and prompts [37]. Since access to health professionals was identified as being important [31], combining technological interventions with access to physicians and other health care professionals may be desirable. It may also be relevant to integrate theory-informed psychological interventions to increase the users’ self-efficacy and motivation to change, such as implementing the Theory of Behavior Change, the Transtheoretical Model of Health Behavior Change, and implementation intentions [38-40]. It would also be relevant to study the stage of change (eg, preparedness vs denial) patients are in and to tailor the interventions accordingly [40]. It would also be interesting to evaluate opioid users’ adherence to behavioral changes in a tailored intervention according to their stage of change, their motivation, and their self-efficacy levels.

The technology holds potential given the high usability and willingness of participants to try these wearables and apps. However, future studies should aim to address some adherence issues surrounding completion rates and reaching out to the most vulnerable groups. The decline in survey completion rates over time, as reported by Hawk et al [24], highlights a challenge in maintaining user engagement, and the issue of homelessness, as discussed by Ahamad et al [25], raises concerns about the reach of such interventions among the most vulnerable populations.

### Strengths and Limitations

This review is important as it explored user perspectives on mHealth for opioid use management and overdose prevention, providing new insights into consumer acceptability in a newly developing field. We focused on emerging wearables and apps that were evaluated over the past 5 years. A limitation is that we did not explore older devices, but our aim was to focus on the latest technology. It should be noted that the findings of this review are limited by the few studies that have been undertaken on the population with OUD during this period. It is necessary to better understand this population’s needs to develop technologically tailored interventions that may best maximize the benefits of assisting with the disorder and preventing fatalities while limiting barriers to using mHealth apps and wearables. More studies are needed to explore future consumer perspectives on mHealth apps for opioid use management. Another limitation is that we did not focus on commercially available apps if they were not evaluated in peer-reviewed medical literature for consumer perspectives. However, our focus was to better understand user experiences; hence, reviewing commercial applications that were not evaluated was not our goal. We also did not focus on efficacy, something that should be further explored alongside mixed methods qualitative research in order to make the best future recommendations.

Here is a list of recommendations for moving this technology forward:

1. Ensure robust privacy measures to address data security concerns, including geolocation tracking and device theft.
2. Provide thorough user training for confident and safe use of mHealth apps and wearables.
3. Integrate more BCTs to potentially increase engagement and positive opioid use management.
4. Provide health care professionals and peer support for enhanced mHealth intervention outcomes.
5. Deliver tailored messages considering participants’ needs and preferences for increased intervention effectiveness.
6. Incorporate behavior change theories for enhanced motivation and self-efficacy.
7. Evaluate participants’ needs and tailor interventions accordingly.
8. Design stigma-reducing mHealth apps and wearables to promote acceptance and help-seeking.
9. Investigate user perspectives to develop tailored mHealth interventions for effective opioid management.

### Conclusion

In conclusion, while mHealth apps and wearables show potential for adoption among patients with OUD, there is a scarcity of studies on consumer perspectives. More research is required to address the specific needs of this population. Common themes in the reviewed studies highlighted the benefits of using mHealth technology to prevent fatalities and improve accessibility to care. However, participants expressed concerns about data privacy and faced technological challenges. To advance mHealth technology for managing OUD, future development should focus on reducing technical barriers, prioritizing patient privacy, and enhancing access to health care professionals. By implementing our 10 recommendations, we can drive progress in health technology, ensuring user-centered design, increased engagement, and improved outcomes for individuals seeking support in managing their OUD.

## Appendix

Multimedia Appendix 1 PRISMA checklist.

## Abbreviations

BCT: behavior change technique
mHealth: mobile health
OUD: opioid use disorder
PRISMA-ScR: Preferred Reporting Items for Systematic Reviews and Meta-Analyses extension for Scoping Reviews

## References

[1] Ghose R, Forati AM, Mantsch JR (2022). Impact of the COVID-19 pandemic on opioid overdose deaths: a spatiotemporal analysis. J Urban Health.

[2] Gomes T, Kitchen SA, Murray R (2021). Measuring the burden of opioid-related mortality in Ontario, Canada, during the COVID-19 pandemic. JAMA Netw Open.

[3] Sakhuja A, Sztajnkrycer M, Vallabhajosyula S, Cheungpasitporn W, Patch R, Jentzer J (2017). National trends and outcomes of cardiac arrest in opioid overdose. Resuscitation.

[4] Parthvi R, Agrawal A, Khanijo S, Tsegaye A, Talwar A (2019). Acute opiate overdose: an update on management strategies in emergency department and critical care unit. Am J Ther.

[5] (2014). Community management of opioid overdose. World Health Organization.

[6] Wermeling DP (2015). Review of naloxone safety for opioid overdose: practical considerations for new technology and expanded public access. Ther Adv Drug Saf.

[7] Nuamah J, Mehta R, Sasangohar F (2020). Technologies for opioid use disorder management: mobile app search and scoping review. JMIR Mhealth Uhealth.

[8] Gullapalli BT, Carreiro S, Chapman BP, Ganesan D, Sjoquist J, Rahman T (2021). OpiTrack: a wearable-based clinical opioid use tracker with temporal convolutional attention networks. Proc ACM Interact Mob Wearable Ubiquitous Technol.

[9] Kanter K, Gallagher R, Eweje F, Lee A, Gordon D, Landy S, Gasior J, Soto-Calderon H, Cronholm PF, Cocchiaro B, Weimer J, Roth A, Lankenau S, Brenner J (2021). Willingness to use a wearable device capable of detecting and reversing overdose among people who use opioids in Philadelphia. Harm Reduct J.

[10] Kulman E, Chapman B, Venkatasubramanian K, Carreiro S (2021). Identifying opioid withdrawal using wearable biosensors. Proc Annu Hawaii Int Conf Syst Sci.

[11] Mahmud MS, Fang H, Wang H, Carreiro S, Boyer E (2018). Automatic detection of opioid intake using wearable biosensor. Int Conf Comput Netw Commun.

[12] Roth AM, Tran NK, Cocchiaro B, Mitchell AK, Schwartz DG, Hensel DJ, Ataiants J, Brenner J, Yahav I, Lankenau SE (2021). Wearable biosensors have the potential to monitor physiological changes associated with opioid overdose among people who use drugs: A proof-of-concept study in a real-world setting. Drug Alcohol Depend.

[13] Salgado García Francisco I, Indic P, Stapp J, Chintha KK, He Z, Brooks JH, Carreiro S, Derefinko KJ (2022). Using wearable technology to detect prescription opioid self-administration. Pain.

[14] Carreiro S, Wittbold K, Indic P, Fang H, Zhang J, Boyer EW (2016). Wearable biosensors to detect physiologic change during opioid use. J Med Toxicol.

[15] Nandakumar R, Gollakota S, Sunshine JE (2019). Opioid overdose detection using smartphones. Sci Transl Med.

[16] Singh R, Lewis B, Chapman B, Carreiro S, Venkatasubramanian K (2019). A machine learning-based approach for collaborative non-adherence detection during opioid abuse surveillance using a wearable biosensor. Biomed Eng Syst Technol Int Jt Conf BIOSTEC Revis Sel Pap.

[17] Chintha KK, Indic P, Chapman B, Boyer EW, Carreiro S (2018). Wearable biosensors to evaluate recurrent opioid toxicity after naloxone administration: a hilbert transform approach. Proc Annu Hawaii Int Conf Syst Sci.

[18] Aglipay M, Wylie JL, Jolly AM (2015). Health research among hard-to-reach people: six degrees of sampling. CMAJ.

[19] Matsuzaki M, Vu QM, Gwadz M, Delaney JAC, Kuo I, Trejo MEP, Cunningham WE, Cunningham CO, Christopoulos K (2018). Perceived access and barriers to care among illicit drug users and hazardous drinkers: findings from the Seek, Test, Treat, and Retain data harmonization initiative (STTR). BMC Public Health.

[20] Bennett AS, Freeman R, Des Jarlais DC, Aronson ID (2020). Reasons people who use opioids do not accept or carry no-cost naloxone: qualitative interview study. JMIR Form Res.

[21] Arun P, Chavan BS, Kaur H (2004). A study of reasons for not seeking treatment for substance abuse in community. Indian J Psychiatry.

[22] Moher David, Shamseer Larissa, Clarke Mike, Ghersi Davina, Liberati Alessandro, Petticrew Mark, Shekelle Paul, Stewart Lesley A, PRISMA-P Group (2015). Preferred reporting items for systematic review and meta-analysis protocols (PRISMA-P) 2015 statement. Syst Rev.

[23] Godersky ME, Klein JW, Merrill JO, Blalock KL, Saxon AJ, Samet JH, Tsui JI (2020). Acceptability and feasibility of a mobile health application for video directly observed therapy of buprenorphine for opioid use disorders in an office-based setting. J Addict Med.

[24] Hawk K, Malicki C, Kinsman J, D'Onofrio G, Taylor A, Venkatesh A (2021). Feasibility and acceptability of electronic administration of patient reported outcomes using mHealth platform in emergency department patients with non-medical opioid use. Addict Sci Clin Pract.

[25] Ahamad K, Dong H, Johnson C, Hyashi K, DeBeck K, Milloy MJ, Wood E (2019). Factors associated with willingness to wear an electronic overdose detection device. Addict Sci Clin Pract.

[26] Eaves ER, Doerry E, Lanzetta SA, Kruithoff KM, Negron K, Dykman K, Thoney O, Harper CC (2023). Applying user-centered design in the development of a supportive mHealth app for women in substance use recovery. Am J Health Promot.

[27] Flickinger TE, Waselewski M, Tabackman A, Huynh J, Hodges J, Otero K, Schorling K, Ingersoll K, Tiouririne NAD, Dillingham R (2022). Communication between patients, peers, and care providers through a mobile health intervention supporting medication-assisted treatment for opioid use disorder. Patient Educ Couns.

[28] Glass JE, Matson TE, Lim C, Hartzler AL, Kimbel K, Lee AK, Beatty T, Parrish R, Caldeiro RM, McWethy AG, Curran GM, Bradley KA (2021). Approaches for implementing app-based digital treatments for drug use disorders into primary care: a qualitative, user-centered design study of patient perspectives. J Med Internet Res.

[29] Hodges J, Waselewski M, Harrington W, Franklin T, Schorling K, Huynh J, Tabackman A, Otero K, Ingersoll K, Tiouririne NAD, Flickinger T, Dillingham R (2022). Six-month outcomes of the HOPE smartphone application designed to support treatment with medications for opioid use disorder and piloted during an early statewide COVID-19 lockdown. Addict Sci Clin Pract.

[30] Langdon KJ, Scherzer C, Ramsey S, Carey K, Rich J, Ranney ML (2021). Feasibility and acceptability of a digital health intervention to promote engagement in and adherence to medication for opioid use disorder. J Subst Abuse Treat.

[31] Magee MR, McNeilage AG, Avery N, Glare P, Ashton-James CE (2021). mHealth interventions to support prescription opioid tapering in patients with chronic pain: qualitative study of patients' perspectives. JMIR Form Res.

[32] Marcu G, Aizen R, Roth AM, Lankenau S, Schwartz DG (2020). Acceptability of smartphone applications for facilitating layperson naloxone administration during opioid overdoses. JAMIA Open.

[33] Tofighi B, Durr M, Marini C, Lewis CF, Lee JD (2022). A mixed-methods evaluation of the feasibility of a medical management-based text messaging intervention combined with buprenorphine in primary care. Subst Abuse.

[34] Waselewski ME, Flickinger TE, Canan C, Harrington W, Franklin T, Otero KN, Huynh J, Waldman ALD, Hilgart M, Ingersoll K, Tiouririne NAT, Dillingham RA (2021). A mobile health app to support patients receiving medication-assisted treatment for opioid use disorder: development and feasibility study. JMIR Form Res.

[35] Zhang M, Ying J, Amron SB, Mahreen Z, Song G, Fung DSS, Smith HE (2019). A smartphone attention bias app for individuals with addictive disorders: feasibility and acceptability study. JMIR Mhealth Uhealth.

[36] Michie S, Wood CE, Johnston M, Abraham C, Francis JJ, Hardeman W (2015). Behaviour change techniques: the development and evaluation of a taxonomic method for reporting and describing behaviour change interventions (a suite of five studies involving consensus methods, randomised controlled trials and analysis of qualitative data). Health Technol Assess.

[37] Michie S, Richardson M, Johnston M, Abraham C, Francis J, Hardeman W, Eccles MP, Cane J, Wood CE (2013). The behavior change technique taxonomy (v1) of 93 hierarchically clustered techniques: building an international consensus for the reporting of behavior change interventions. Ann Behav Med.

[38] Tapera R, Mbongwe B, Mhaka-Mutepfa M, Lord A, Phaladze NA, Zetola NM (2020). The theory of planned behavior as a behavior change model for tobacco control strategies among adolescents in Botswana. PLoS One.

[39] Orbell S, Hodgkins S, Sheeran P (1997). Implementation intentions and the theory of planned behavior. Pers Soc Psychol Bull.

[40] Prochaska JO, Velicer WF (1997). The transtheoretical model of health behavior change. Am J Health Promot.

